# Chimeric Antigen Receptor T‐Cell Therapy and Autoimmune Diseases in the Nervous System

**DOI:** 10.1002/iid3.70298

**Published:** 2025-11-10

**Authors:** Shun‐yu Yao, Miao‐qiao Du, Huan Yang, Qiu‐ming Zeng, Hao Zhou, Xiuli Zhang, Sugimoto Kazuo, Jia Liu, Lan‐xin Lin, Xu‐hui Kang, Dai‐yi Jiang, Yong Peng

**Affiliations:** ^1^ Department of Neurology Affiliated First Hospital of Hunan Traditional Chinese Medical College Zhuzhou Hunan China; ^2^ Department of Neurology Affiliated Provincial Traditional Chinese Medical Hospital of Hunan University of Chinese Medicine Zhuzhou Hunan China; ^3^ Department of Neurology Xiangya Hospital, Central South University Changsha Hunan China; ^4^ Science and Technology Innovation Center Hunan University of Chinese Medicine Changsha China; ^5^ Department of Neurology, Dongzhimen Hospital Beijing University of Chinese Medicine Beijing China; ^6^ Institute for Brain Disorders Beijing University of Chinese Medicine Beijing China

**Keywords:** autoimmune diseases, autoimmune encephalitis, CAR‐T therapy, Guillain–Barré syndrome, multiple sclerosis, Myasthenia Gravis, myelin oligodendrocyte glycoprotein antibody‐associated disease, neuromyelitis optica spectrum disorder

## Abstract

**Introduction:**

Chimeric antigen receptor T‐cell (CAR‐T) therapy, a revolutionary immunotherapy originally developed for hematologic malignancies, has recently gained attention for its potential in treating autoimmune diseases. Increasing evidence suggests that CAR‐T cells can precisely target pathogenic immune populations, offering durable remission and immune homeostasis restoration in neuroimmunological disorders such as myasthenia gravis (MG), neuromyelitis optica spectrum disorder (NMOSD), and multiple sclerosis (MS).

**Methods:**

Relevant publications and clinical trial data up to September 2025 were systematically reviewed to summarize the mechanisms, therapeutic targets, safety profiles, and translational applications of CAR‐T therapy in autoimmune diseases of the nervous system.

**Results:**

Preclinical and early clinical studies demonstrate that CD19‐ and BCMA‐directed CAR‐T therapies effectively deplete autoreactive B cells with significant symptom improvement and minimal cytokine release syndrome or neurotoxicity. Novel constructs such as chimeric autoantibody receptor (CAAR)‐T and CAR‐regulatory T (CAR‐Treg) cells enhance specificity and immune tolerance. Innovations including allogeneic “off‐the‐shelf” CAR‐T, in vivo CAR engineering, and CRISPR‐based safety switches further optimize therapeutic potential and accessibility.

**Conclusion:**

CAR‐T therapy represents a promising frontier for refractory neuroautoimmune diseases. By precisely modulating immune networks, it offers a pathway toward long‐term remission and personalized immunotherapy in clinical neuroimmunology.

## Summary

- **Success of CAR‐T therapy in cancer immunotherapy**: CAR‐T therapy has shown significant efficacy in treating various hematological malignancies, marking a revolutionary advancement in cancer immunotherapy.
- **Potential of CAR‐T in autoimmune diseases**: This section discusses the fundamental principles of immune diseases and similarities between immune system disorders and tumors in terms of CAR‐T therapy. Recent immunological research has indicated that CAR‐T therapy holds potential for application in autoimmune diseases.
- **Application of CAR‐T in neuroimmunological diseases**: CAR‐T therapy development for neuroimmunological disorders is progressively advancing and encompasses seven treatment‐resistant conditions. This section discusses the therapeutic mechanisms, available animal models, and clinical findings. CAR‐T therapy has shown promising results, especially in refractory case treatment.
- **Immune modulation mechanism:** CAR T cells are engineered to target cancer or dysfunctional immune cells, thereby regulating the immune response. CAR T cells target pathogenic B, T, and other immune cell populations in autoimmune diseases, thereby suppressing excessive immune reactions.
- **Challenges and future development:** Although CAR‐T therapy holds significant promise for treating autoimmune diseases, issues such as immune tolerance, cytokine release syndrome, and neurotoxicity persist. Future research, including combination therapies and optimization of CAR‐T safety profiles, may enhance therapeutic efficacy.

## Graphical abstract

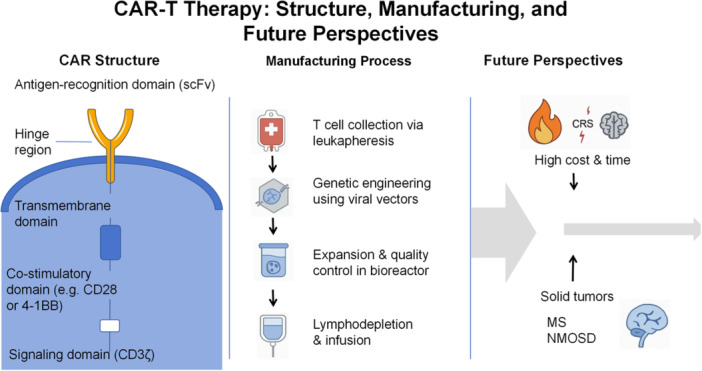

**Abbreviations**
AChR: acetylcholine receptor
AE: autoimmune encephalitis
AIDs: autoimmune diseases
ALL: acute lymphoblastic leukemia
ANCA: anti‐neutrophil cytoplasmic antibody
AQP4: aquaporin‐4
BCMA: B‐cell maturation antigen
B‐ALL: B‐cell acute lymphoblastic leukemia
CAAR‐T: chimeric autoantibody receptor T‐cell
CAR: chimeric antigen receptor
CAR‐T: chimeric antigen receptor T‐cell
CNS: central nervous system
CRS: cytokine release syndrome
CSF: cerebrospinal fluid
CTLA‐4: cytotoxic T‐lymphocyte‐associated protein
DLBCL: diffuse large B‐cell lymphoma
DMTs: disease‐modifying therapies
EAE: experimental autoimmune encephalomyelitis
FL: follicular lymphoma
GBS: Guillain–Barré syndrome
ICANS: immune effector cell‐associated neurotoxicity syndrome
IIM: idiopathic inflammatory myopathy
IL‐6: interleukin‐6
IVIG: intravenous immunoglobulin
MG: myasthenia gravis
MHC: major histocompatibility complex
MM: multiple myeloma
MOGAD: myelin oligodendrocyte glycoprotein antibody‐associated disease
MS: multiple sclerosis
MuSK: muscle‐specific kinase
NMOSD: neuromyelitis optica spectrum disorder
NS: nervous system
RA: rheumatoid arthritis
rCAR‐T: RNA‐engineered chimeric antigen receptor T‐cell
SLE: systemic lupus erythematosus
Tregs: regulatory T cells

## 1 Introduction

Chimeric antigen receptor T‐cell (CAR‐T) therapy has revolutionized cancer treatment, particularly for hematologic malignancies such as B‐cell acute lymphoblastic leukemia and non‐Hodgkin lymphoma [1]. This groundbreaking approach, involving genetic T cell modification to target cancer‐specific antigens, has prompted interest in its potential for treating autoimmune diseases [2].

Autoimmune disorders, including rheumatoid arthritis (RA), systemic lupus erythematosus (SLE), and type 1 diabetes (T1D), are characterized by excessive immune responses that damage tissues. Current treatments often involve broad‐spectrum immunosuppressive drugs, which alleviate symptoms but fail to provide long‐term disease control and can cause severe side effects [3]. Therefore, targeted therapies are urgently needed [1].

Although CAR‐T therapy has shown significant success in treating cancer, its use in autoimmune diseases remains largely experimental [4]. Under autoimmune conditions, CAR T cells can be engineered to target specific immune cells, such as pathogenic B cells or autoreactive T cells, instead of cancerous cells [5]. This precision‐based approach holds the potential for effective treatment with fewer side effects than conventional therapies [3].

This review explores the mechanisms and potential applications of CAR‐T therapy in autoimmune neuroimmunological disorders, contrasting its application in cancer with its evolving use in autoimmune diseases of the nervous system (NS). In addition, we examined ongoing clinical trials and revealed future research directions, emphasizing the transformative potential of CAR T cells for immune system modulation in the treatment of autoimmune disorders.

## 2 CAR‐T Therapy Overview

### 2.1 Definition

CAR‐T therapy involves genetically modifying the T cells of a patient to express synthetic receptors (CARs) that target specific antigens. This technique bypasses major histocompatibility complex (MHC) restrictions, enabling CAR T cells to directly recognize and attack target cells without requiring antigen presentation [6, 7]. CAR typically includes an extracellular domain for antigen recognition, a transmembrane segment, and an intracellular domain for T cell activation [8].

Recent advancements in CAR‐T design, such as costimulatory domain incorporation, including CD28 and 4‐1BB, have improved T cell proliferation and persistence, enhancing the efficacy of therapy [9]. These innovations have been instrumental in the success of CAR‐T therapy in hematological cancers [10].

### 2.2 CAR‐T Manufacturing Process

The CAR‐T manufacturing process involves several critical steps to ensure the safety and effectiveness [11].

#### 2.2.1 T Cell Collection

T cells are harvested from the blood of a patient through leukapheresis, providing a foundation for CAR‐T therapy [11].

#### 2.2.2 Genetic Engineering

The T cells are modified using viral vectors to express CARs, allowing them to target specific antigens such as CD19 [12, 13]. This modification ensured that CAR‐T cells bypassed the need for MHC presentation.

#### 2.2.3 Expansion and Quality Control

Modified T cells are expanded ex vivo under controlled conditions, ensuring that they meet predefined safety and potency criteria [14]. This step is crucial to ensure the efficacy of CAR‐T infusion into patients [15].

#### 2.2.4 Patient Conditioning and Infusion

Before infusion, patients undergo lymphodepleting chemotherapy to create a favorable environment for CAR‐T cells to expand and function effectively [16]. After infusion, CAR‐T cells seek and eliminate target cells that exhibit long‐term memory‐like properties [7].

### 2.3 Challenges and Future Directions

Despite its success in hematological malignancies, CAR‐T therapy faces challenges, including serious side effects such as cytokine release syndrome (CRS) and neurotoxicity, which require careful patient monitoring [17]. However, high production costs and long manufacturing times limit their accessibility. However, efforts to develop universally applicable CAR‐T cells sourced from healthy individuals may reduce costs and improve accessibility [18]. Recent research has focused on expanding the applications of CAR‐T cells to solid tumors and autoimmune diseases, with promising preliminary findings revealing their potential efficacy in treating conditions such as multiple sclerosis (MS) and neuromyelitis optica spectrum disorder (NMOSD) [10]. With technological advances, CAR‐T therapy may provide novel targeted treatment options for autoimmune neurological diseases, with implications for precision medicine [3].

## 3 Shared Mechanisms of CAR‐T Therapy in Autoimmune Diseases and Cancer

### 3.1 Immunological Overlap Between Cancer and Autoimmunity

Although cancer and autoimmune diseases appear fundamentally different, cancer arising from immune evasion and autoimmunity from immune overactivation share a core mechanism: immune system dysregulation [19, 20]. These findings indicate that modulating immune responses, either to restore tolerance or reinvigorate antitumor activity, may provide a dual‐purpose therapeutic strategy [20, 21].

### 3.2 Bridging Therapeutic Gaps Through Immune Checkpoints

Historically, cancer and autoimmune diseases have been treated independently. However, the discovery of immune checkpoints (PD‐1 and CTLA‐4) and their inhibitors has provided unified therapeutic strategies in these fields [19, 22]. Immune checkpoint blockade, once cancer‐specific, is now being studied for autoimmune modulation [21].

### 3.3 Role of Chronic Inflammation and Tissue Microenvironment

Chronic inflammation is a hallmark of cancer and autoimmune diseases [23]. Inflammatory microenvironments marked by hypoxia, macrophage infiltration, and T‐cell activation are observed in both tumor tissues and autoimmune lesions [24, 25]. In addition, gut microbiota dysbiosis influences immune homeostasis and may contribute to the pathogenesis of both diseases [21].

### 3.4 Cancer–Autoimmunity Bidirectional Relationship

Autoimmune diseases can predispose individuals to cancer development, as seen in patients with autoimmune rheumatic diseases [4, 26]. Conversely, cancer immunotherapy can trigger autoimmune‐like complications, further blurring the line between these two conditions [22]. This intersection has accelerated interest in CAR‐T therapy as a bridge between oncology and immunology [27].

### 3.5 CAR‐T Design for Cancer and Autoimmunity

In cancer, CAR‐T cells are tailored to target tumor antigens, leading to direct cytotoxicity against malignant cells [28]. CAR‐T therapy aims to eliminate autoreactive immune cells in autoimmune diseases. For example, CD20‐targeting CAR‐T cells have shown efficacy in both B‐cell lymphomas and RA [29]. Another novel target is CXCR3, a chemokine receptor involved in immune cell trafficking and inflammation in various diseases [30, 31].

### 3.6 Dual Role of the CXCR3‐CXCL9/10 Axis

In RA, elevated CXCR3 expression and its ligands (CXCL9/10) correlate with disease severity [32, 33]. Similarly, the CXCR3 pathway contributes to lupus nephritis and IBD [34]. In cancer, this axis supports T cell infiltration into tumors, thereby enhancing antitumor response. In addition, CXCL4/4L1 ligands suppress angiogenesis and tumor metastasis, reinforcing the dual relevance of this pathway [35].

### 3.7 Targeting interleukin‐6 (IL‐6) and COX‐2

IL‐6 is central to inflammation in both cancer and autoimmune diseases. It promotes Th17 cell differentiation and chronic inflammation in patients with RA [28]. Approved IL‐6 inhibitors for RA are currently being investigated for use in cancers such as prostate cancer, where IL‐6–STAT3 signaling promotes tumor growth. Similarly, COX‐2 inhibitors (such as celecoxib) used to treat arthritis show antitumor effects despite cardiovascular risks [36, 37].

### 3.8 CD6–CD318 Axis: A Promising Therapeutic Target

The CD6–CD318 axis modulates Th1/Th17 differentiation and enhances T/NK cell cytotoxicity. Preclinical studies have shown that this pathway can be dual‐targeted to suppress autoimmune inflammation and promote antitumor immunity [28].

### 3.9 Limitations and Safety Considerations

**Figure 1:**
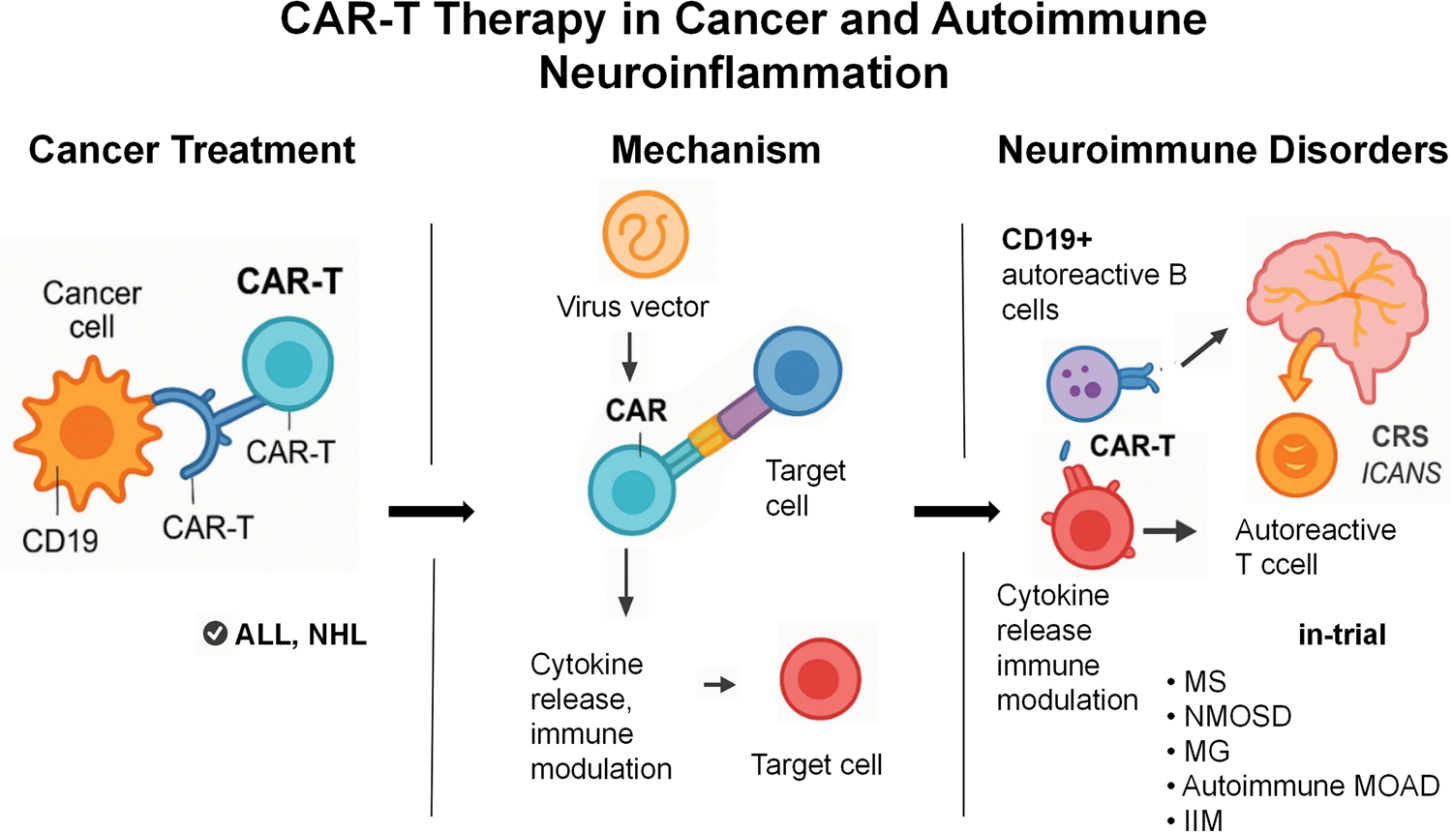
Overview of CAR‐T therapy: structure, manufacturing process, and future perspectives. The left panel shows the structure of a second‐generation CAR, including antigen recognition (scFv), hinge, transmembrane, costimulatory (CD28 or 4‐1BB), and signaling (CD3ζ) domains. The middle panel illustrates the CAR‐T manufacturing process, consisting of T cell collection, genetic modification, ex vivo expansion, and patient infusion. The right panel highlights current challenges such as cytokine release syndrome (CRS), neurotoxicity (ICANS), and high production costs, while pointing to promising future applications in solid tumors and autoimmune neurological disorders such as MS and NMOSD.

Despite its promise, CAR‐T therapy can induce autoimmune‐like syndromes in patients with cancer, necessitating careful risk‐benefit assessment [28]. New strategies, such as the use of regulatory CAR‐T and chimeric autoantibody receptor (CAAR) T cells, are being developed to enhance safety and specificity (Figure 1) [4].

## 4 Possible CAR‐T Therapy Mechanism in Autoimmune Disease

The potential mechanisms of CAR‐T therapy, which was initially successful in cancer treatment, are now being investigated as a novel therapeutic approach for autoimmune diseases [38]. This therapy involves engineering T cells to specifically target and eliminate pathological antigens, thereby providing a precise alternative to conventional treatment for autoimmune diseases. CAR‐T therapy is characterized by fewer side effects and longer‐lasting therapeutic effects than traditional immunosuppressive therapies [39]. These T cells modified with CARs secrete cytotoxic substances such as perforin and granzymes to target autoreactive immune cells and direct both effector and regulatory T cells (Tregs) into the autoimmune milieu, thereby enhancing immune regulation and promoting self‐tolerance restoration [40]. The multidimensional action of CAR‐T therapy makes it a promising approach for treating autoimmune diseases [4].

The mechanisms through which CAR‐T therapy affects autoimmune diseases can be classified into three key approaches:

### 4.1 Cytotoxic Activity Against Target Cells

CAR‐T cells achieve cytotoxic function by recognizing and directly eliminating target cells expressing specific antigens. These target cells are often autoreactive immune cells, especially B cells, which produce pathogenic autoantibodies. For instance, in SLE, CD19‐targeted CAR‐T cells are used to reduce the number of CD19^+^ B cells responsible for the production of harmful autoantibodies. Upon infusion, these CAR‐T cells expand in the body of the patient and specifically target and deplete B cells, thus reducing the inflammatory responses and immune dysregulation characteristic of SLE [3, 4]. This approach provides more precise and targeted therapy than the broad range of immunosuppressive agents typically used for treating autoimmune diseases [38].

### 4.2 CAAR‐T

CAAR‐T cells represent an advanced form of CAR‐T therapy specifically designed to target B cells that secrete autoantibodies. For example, in the case of myasthenia gravis (MG), muscle‐specific kinase (MuSK) CAAR‐T cells are engineered to target and eliminate B cells that produce MuSK antibodies. The extracellular domain of the MuSK antigen is incorporated into the CAR, allowing these cells to specifically target and destroy the pathogenic B cells responsible for autoimmune damage in patients with MG. By selectively depleting these B cells, CAAR‐T cells can reduce disease progression and improve clinical outcomes in patients with MG [41].

### 4.3 CAR‐Regulatory T Cells (CAR‐Tregs)

CAR‐Treg therapy entails modifying Tregs to direct and regulate the activity of autoreactive immune cells, thereby reestablishing immune balance. In autoimmune diseases, such as T1D, CAR‐Treg therapy uses second‐generation CAR constructs containing insulin‐specific single‐chain variable fragments (scFvs) and the Foxp3 sequence, a key marker for Tregs. CAR‐Tregs are derived from CD4^+^ T effector cells and reprogrammed into insulin‐specific Tregs (CAR‐cTregs). CAR‐Tregs help mitigate immune‐mediated tissue destruction and restore self‐tolerance by targeting autoreactive T cells and other immune cells involved in autoimmune damage. This approach has significant potential for treating diseases driven by immune dysregulation and could provide a more targeted therapeutic option than conventional treatments [42].

In summary, CAR‐T therapy for autoimmune diseases leverages genetically modified immune cells to specifically target and modulate the immune system [3]. This approach provides a more specific, effective, and long‐lasting alternative to traditional treatments that often rely on broad immunosuppressive strategies, which fail to address the root causes of autoimmune diseases. By targeting autoreactive immune cells and enhancing immune regulation through engineered Tregs, CARfigure‐T therapy could provide a tailored and efficient therapeutic option for autoimmune diseases in the near future [41].

#### 4.3.1 Rationale for CD19 Targeting and Comparison With CD20 and BCMA

Most CAR‐T therapies for autoimmune and neuroimmunological diseases have been directed against CD19, a pan‐B‐cell marker expressed from the early stages of B‐cell development through most stages of differentiation, except in plasma cells. CD19 is consistently expressed in autoreactive B‐cell populations, including memory B cells and plasmablasts, which are central to autoantibody production and the pathogenesis of disease. This broad and stable expression pattern makes CD19 an ideal therapeutic target, explaining why it is the dominant antigen in both hematological malignancy and autoimmune disease trials [43, 44].

### 4.4 Comparison With CD20

CD20 is another well‐established B‐cell marker extensively targeted by monoclonal antibodies such as rituximab. However, CD20 expression begins at the pre‐B stage and is lost upon differentiation into the plasma cells. Consequently, CD20‐directed therapies eliminate circulating mature B cells but spare plasmablasts and plasma cells that continue secreting pathogenic autoantibodies. In contrast, CD19 is retained on plasmablasts and subsets of plasma cells, enabling a more complete depletion of autoreactive clones [27, 45]. This distinction may account for the more notable and durable immunomodulation observed with CD19 CAR‐T therapy than with anti‐CD20 approaches.

### 4.5 Comparison With BCMA

B‐cell maturation antigen (BCMA, also known as TNFRSF17) is selectively expressed on plasma cells and plasmablasts, including long‐lived plasma cells that escape CD19‐ or CD20‐targeted therapy. BCMA‐directed CAR‐T therapy provides a complementary approach by directly depleting autoantibody‐secreting cells that sustain chronic disease activity. Preclinical and clinical evidence has revealed that BCMA CAR‐T is highly effective in conditions dominated by pathogenic plasma cells, such as SLE and NMOSD [46, 47].

In summary, CD19 remains the predominant target because of its broad expression across the B‐cell lineage and its ability to deplete both memory B cells and plasmablasts. CD20 therapies are limited by their inability to eliminate plasma cells, whereas BCMA‐targeting CAR‐T provides an important complementary strategy by specifically removing long‐lived plasma cells that escape CD19 depletion. The optimal therapeutic approach may depend on the disease context, and future studies should explore whether sequential or combined targeting of CD19 and BCMA yields synergistic efficacy in complex neuroimmune disorders [48].

## 5 Role of CAR‐T in Treating Autoimmune Diseases in the NS

### 5.1 MG

#### 5.1.1 Rationale

MG is an autoimmune condition characterized by compromised neuromuscular signaling due to autoantibodies directed against acetylcholine receptors (AChRs) or MuSK [49]. Conventional therapies, including corticosteroids and wide range of immunosuppressants, frequently have notable adverse effects and inconsistent effectiveness, highlighting the need for precise treatment approaches. Recent progress in CAR‐T therapy has shown notable promise in treating MG [49, 50]. The pathogenesis involves pathogenic plasma cells and memory B cells, making CD19 and B‐cell maturation antigen (BCMA) attractive CAR targets [51].

#### 5.1.2 CD19 CAR‐T in MG

CD19‐targeted CAR‐T therapy has emerged as a promising strategy in both preclinical and clinical settings. In murine models of MG, CD19‐CAR‐T treatment resulted in a notable reduction in AChR‐specific autoantibodies and improved muscle strength [50]. In clinical applications, anti‐CD19 CAR T cells induce sustained remission in patients with refractory MG by depleting autoreactive B cells and reducing circulating autoantibodies [52]. Haghika et al. highlighted the utility of testing different CAR‐T constructs under the same conditions by treating a single patient with refractory MG using the CD19 approach [49]. Self‐resolving transaminitis was the only adverse event reported during the short follow‐up period (62 days), and clinical improvements in Besinger disease activity and quantitative MG scores were observed. Immunosuppression (through 10 mg prednisolone administration) was continued at the time of reporting, with the intent to withdraw at follow‐up. Single‐cell RNA sequencing of immune cell populations from treated patients identified proliferating cytotoxic‐like CD8^+^ T cell clones as key effectors in reversing autoimmunity [52]. Phase 1 trials using autologous RNA CAR‐T (Descarte‐08) revealed a safe and clinically meaningful reduction in MG severity for up to 9 months [53]. CRS was predominantly low‐grade, and no immune effector cell‐related neurotoxicity syndrome (ICANS) was reported [53].

#### 5.1.3 BCMA CAR‐T in MG

In addition to CD19 targeting, BCMA‐specific CAR‐T cells have shown encouraging outcomes in the management of MG [54]. Granit et al. reported the first study using rCAR‐T therapy in individuals with MG using RNA to improve the safety profile of CAR‐T. This study revealed that the temporary, nonreplicable influence of mRNA would confer predictable pharmacokinetics and, consequently, a favorable safety profile, with no requirement for lymphodepleting conditioning, compared to the standard DNA approach. The trial (NCT04146051) was a prospective, multicenter, open‐label, phase 1b/2a study of Descartes‐08, an autologous anti‐BCMA rCAR‐T therapy. A dose escalation protocol was followed (three patients received three doses, and 11 were planned to receive six doses). The interim results included 16 adult patients with generalized treatment‐refractory MG and a Myasthenia Gravis Activities of Daily Living score of ≥ 6. Almost all patients (13/14) were seropositive (11/14 acetylcholine receptor antibody‐positive and 2/14 anti‐MuSK antibody‐positive). Treatment was completed as planned in 12 patients and was safe and well tolerated, showing clinically meaningful decreases in the Myasthenia Gravis Severity Scale after up to 9 months of follow‐up period. Although antibody reversal was not observed, acetylcholine receptor antibody levels were reduced. However, MuSK titers were not reduced, and rCAR‐T cells were present in the peripheral blood at 1–2 h post‐infusion, but not at later time points. No dose‐limiting toxicity, CRS, or neurotoxicity was reported, with withdrawals cited as being due to one patient experiencing urticaria requiring intravenous steroids (in the dose‐escalation group), which was related to rCAR‐T therapy, and one withdrew for personal reasons [53]. A case series of two patients with refractory MG treated with BCMA–CAR‐T showed sustained clinical benefits and autoantibody depletion over 18 months, with favorable safety and no high‐grade CRS/ICANS [52]. Bispecific BCMA/CD19 CAR‐T cells achieved symptom disappearance and AChR antibodies in a patient with refractory MG [55].

#### 5.1.4 CAAR‐T in MG

MuSK‐specific CAR‐T cells represent an innovative therapeutic approach. Anti‐MuSK CAAR‐T cells have been engineered to selectively target MuSK autoantibody‐producing B cells and exhibit high specificity and cytotoxicity in preclinical studies [41]. These encouraging preclinical results prompted the launch of clinical trials, including the Descartes‐08 CAR‐T cell trial (NCT04146051), aimed at assessing the safety and effectiveness of MuSK‐targeted CAR‐T cells in patients with generalized MG. Clinical data are pending.

Emerging therapies, including B cell‐specific monoclonal antibody‐siRNA conjugates [56] and mesenchymal stem cell therapy [57], complement the growing repertoire of immunomodulatory treatments. These targeted therapies provide multiple benefits compared to conventional immunosuppressants, such as a more rapid onset of action, diminished side effects, and sustained long‐term disease remission [58].

In summary, CAR‐T therapy is a transformative approach for managing MG [59]. By precisely targeting pathogenic B cells and modulating immune responses, CAR‐T therapies have the potential to revolutionize treatment outcomes for patients with MG. Ongoing clinical trials are essential for refining these approaches, optimizing their efficacy, and expanding their applicability to other autoimmune neuromuscular disorders [50, 52].

### 5.2 NMOSD

#### 5.2.1 Rationale

NMOSD, a rare yet serious autoimmune condition affecting the central nervous system (CNS), is characterized by repeated episodes of inflammation and demyelination, primarily affecting the optic nerves and spinal cord [60]. The disease is often associated with autoantibodies targeting aquaporin‐4 (AQP4‐IgG), leading to complement activation and astrocyte damage. Clinically, NMOSD may lead to significant disability, such as blindness and paralysis, significantly affecting patient quality of life [61]. Current treatment strategies primarily focus on immunosuppression using corticosteroids, azathioprine, rituximab and eculizumab. Although these treatments can reduce relapse rates and disease progression, they are often associated with limited efficacy in refractory cases, long‐term toxicity, and a heightened risk of infection due to broad immunosuppression [62].

#### 5.2.2 BCMA‐Targeted CAR‐T in NMOSD

Recent advancements in CAR‐T therapy have shown promise in addressing these challenges by providing a targeted and precise immunotherapeutic approach. Interim results from 12 patients with refractory NMOSD treated with autologous BCMA CAR‐T therapy (NCT04561557) showed an improvement in the clinical examination score, whereas 11 out of 12 remained in remission (absence of relapse and freedom from immunosuppression) at a median follow‐up of 5.5 months. AQP4‐IgG antibody reversal was observed in 70% of patients. One patient whose AQP4‐IgG levels increased after an initial decrease was the only patient who relapsed at 14 months. Improvements were observed in clinical examinations, visual function, bowel and bladder function, quality of life, and ambulation. A dose escalation approach was used, with three patients receiving half the dose, with cyclophosphamide and fludarabine lymphodepletion. CAR‐T‐cell expansion occurred maximally by 10 days, and persistence reduced over follow‐up, with detection at 6 months observed in one of six patients, whereas BCMA levels were significantly reduced at 1 month but returned to baseline by month 6. The single patient who relapsed received a lower dose of CAR‐T cells and, at the time of relapse, had low CAR‐T cell levels and an increased AQP4‐IgG level. All patients experienced expected hematological toxicity (anemia and leukopenia) and grade 1 or 2 CRS, whereas 7 of 12 patients developed an infection, and a minority developed transient gastrointestinal disturbances [63].

An ongoing phase I clinical trial has indicated the safety and benefits of anti‐BCMA CAR‐T cells in 12 patients with AQP4‐IgG seropositive NMOSD. Case reports involving two individuals with progressive MS and one patient with stiff‐person syndrome showed a manageable safety profile following treatment with anti‐CD19 CAR‐T cells. Recruitment commenced for two larger studies on MS, and a phase I open‐label basket study is underway to evaluate BCMA‐directed CAR‐T cells in various antibody‐associated inflammatory diseases, including MOG‐associated diseases. Preclinical research on NMDA receptor antibody autoimmune encephalitis (AE) treated with CAAR‐T cells has generated promising data [64].

Single‐cell analyses revealed that the primary effectors were proliferating cytotoxic‐like CD8^+^ CAR‐T cells, which successfully traversed the blood–brain and blood–cerebrospinal fluid (CSF) barriers to eradicate plasmablasts and plasma cells within the CSF [63], suggesting that CAR‐T cells can target immune cells within the CNS while minimizing off‐target effects, differentiating them from conventional therapies. Here, we performed single‐cell multiomics sequencing of paired CSF and blood samples from patients with NMOSD treated with anti‐BCMA CAR‐T cells. Proliferating cytotoxic‐like CD8^+^ CAR‐T clones have been identified as the primary effectors of autoimmunity. Anti‐BCMA CAR T cells with enhanced chemotactic features efficiently cross the blood–CSF barrier, eliminate plasmablasts and plasma cells in the CSF, and suppress neuroinflammation. The CD44‐expressing early memory phenotype in infusion products is potentially associated with CAR‐T cell persistence in autoimmunity. Moreover, CAR‐T cells from patients with NMOSD display distinctive features of suppressed cytotoxicity compared to those from patients with hematological malignancies [60].

These findings have opened new avenues for precision medicine approaches in NMOSD, including the development of anti‐AQP4 CAAR‐T cells that specifically eliminate autoantibody‐producing plasma cells [65]. This strategy aligns with the treat‐to‐target principle and provides a tailored long‐term therapeutic option for patients with refractory NMOSD. Current ongoing clinical trials further validate this potential, such as studies investigating BAFFR CAR‐T cells (NCT06561009) and universal CAR‐T cells targeting BCMA (NCT06633042), which sought to assess the safety and effectiveness of CAR‐T therapy in refractory AQP4‐IgG‐positive NMOSD, expanding upon prior findings that indicate a promising safety record and fewer adverse events, such as diminished risks of cytokine release syndrome and neurotoxicity [54].

#### 5.2.3 CD19 CAR‐T Data

CAR‐T applications in NMOSD remain limited, with large trials still pending (Smith, 2024). The CD19^+^ precursors of NMOSD antibodies could be targeted, but CNS plasma cells may escape CD19 targeting [64].

Evidence of the benefits of CAR‐T therapy in individuals with CNS‐directed autoimmunity remains minimal. However, multicenter controlled clinical trials with manageable safety profiles appear feasible and are warranted because of promising case experiences [64]. By synthesizing these clinical and preclinical findings, CAR‐T therapy has established itself as a pioneering immunotherapeutic strategy for NMOSD, providing notable clinical advantages and the prospect of sustained remission in patients. CAR T cells may establish a new paradigm for managing NMOSD and other autoimmune CNS diseases [66].

### 5.3 MS

#### 5.3.1 Pathogenesis and Rationale

The NS is a sophisticated network that governs and harmonizes body functions. The NS encompasses the CNS, which consists of the brain, spinal cord, and the peripheral nervous system (PNS), linking the CNS to the remainder of the body [67]. NS disorders, particularly autoimmune diseases such as MS, pose significant therapeutic challenges. MS is a chronic inflammatory condition marked by immune‐mediated damage to the myelin sheath and axons, resulting in progressive neurological impairment. Although there have been advancements in the understanding of its pathogenesis, MS remains uncured, and current therapies predominantly aim to manage symptoms, decrease relapses, and slow disease progression [68].

The clinical management of MS poses several challenges. Traditional disease‐modifying therapies, such as interferons, glatiramer acetate, and monoclonal antibodies targeting B cells (such as ocrelizumab and rituximab), can reduce disease activity but are associated with significant limitations, including incomplete efficacy, frequent relapses, and risks of adverse effects, such as infections and malignancies [69]. Advanced treatments, including hematopoietic stem cell transplantation, provide the potential for sustained remission but entail significant risks, such as therapy‐associated mortality and significant immunosuppression. These limitations highlight the urgent need for innovative approaches that can effectively target the complex immune mechanisms underlying MS, while minimizing off‐target effects [67].

This is highly relevant for patients with MS, considering the pre‐existing knowledge of the underlying disease mechanism and the role of Epstein–Barr virus infection and persistent autoreactive B cells, a target for which refractory EBV‐related lymphomas are successfully treated with CAR‐T cells [65].

#### 5.3.2 CD19 CAR‐T in MS

Recent developments in CAR‐T therapy have shown promise in addressing MS, especially its progressive variants. A groundbreaking clinical study provided the first evidence of CD19‐targeted CAR‐T therapy safety and potential efficacy in two patients with progressive MS [70, 71]. CD19 CAR‐T cell administration was well tolerated, with no occurrence of ICANS, even though CAR‐T cells were identified in the CSF. A significant reduction in intrathecal antibody production was observed in one patient, which was sustained until day 64 post‐infusion. This suggests that CAR‐T cells can modulate the pathogenic immune response in MS without inducing neurotoxicity by expanding and targeting depletion of CD19^+^ B cells in the CNS. A separate case showed dramatic improvement in spasticity and mobility with low‐grade CRS but no ICANS [72].

Ongoing clinical trials, such as the evaluation of CC‐97540 in relapsing or progressive MS (NCT06220201), are expected to provide valuable insights into the safety, tolerability, and efficacy of advanced cell‐based therapies. As research progresses, CAR‐T therapy holds promise as a transformative treatment for MS as well as a blueprint for ddressing other autoimmune diseases of the NS. Larger clinical trials are required to validate these findings, optimize protocols, and establish CAR‐T therapy as a viable option for the management of MS [65].

#### 5.3.3 BCMA and CAAR‐T in MS

BCMA‐directed CAR‐T for other antibody‐mediated CNS diseases is under investigation, and basket trials include MOG‐AD but are not yet specific for MS [73]. CAAR‐T cells targeting specific autoreactive B cells in MS are still in the preclinical stage [74].

#### 5.3.4 Preclinical Research

Preclinical trials in neurological disorders, in the experimental autoimmune encephalomyelitis (EAE) animal model of MS, and mouse models of MuSK MG and anti‐*N*‐methyl‐d‐aspartate receptor encephalitis have targeted specific antibodies instead of the entire population of cells expressing CD19 or BCMA with sustained treatment effects [41, 75, 76, 77]. CAR‐T therapy targeting myelin basic protein, myelin oligodendrocyte glycoprotein (in EAE), and MuSK IgG (in MuSK MG) has resulted in sustained treatment effects. Fransson et al. reported the creation of an immunotolerant environment after treatment when symptom‐free mice were rechallenged with EAE [76]. Targeting MuSK had a similar efficacy to CD19 CAR‐T therapy but resulted in specific MuSK B‐cell depletion without reducing total B cells or IgG levels, and freedom from off‐target effects [41]. Gupta et al. used anti‐CD19 CAR‐T cells in mice models, which showed improvement in clinical scores and lymphocyte infiltration in the tissue, in contrast to previous data that showed exacerbation of EAE after anti‐CD19 CAR‐T therapy.

Preclinical research corroborates these results, showing that anti‐CD19 CAR‐T cells can mitigate EAE, a commonly used animal model of MS, by depleting B cells in both peripheral tissues and the CNS [78]. Furthermore, Tregs expressing myelin‐specific CARs have been effective in inhibiting autoimmune reactions in EAE models, presenting another potential approach for cell‐based therapies [75]. However, translating preclinical findings into therapeutic approaches for humans poses a significant challenge, largely because of the intricate interactions among immune cells in the pathogenesis of MS, including B cells, T cells, and elements of the innate immune system [79, 80].

In addition to CD19‐targeted approaches, alternative strategies are being investigated to further enhance their therapeutic efficacy. Liver‐directed gene therapy has shown promise in inducing antigen‐specific Treg cells and effectively reversing disease progression in EAE models [81]. Other cell therapeutic strategies include myelin‐forming oligodendrocyte replacement, hematopoietic stem cell transplantation, and mobilization of endogenous stem cells, all of which aim to restore integrity and function [80].

This growing body of evidence underscores the potential of CAR‐T therapy to revolutionize the treatment of MS, providing hope for patients with limited therapeutic options and paving the way for novel interventions in neuroimmunology (Table 1).

**Table 1:** Comparative analysis of CAR‐T targets in MG, NMOSD, and MS: mechanistic rationale, clinical utility, safety, and limitations.

Disease	Target	Clinical application	Mechanistic advantage	Major limitation	Safety profile (CRS/ICANS)	References
MG	CD19	Phase 1 trial (Descartes‐08) completed in generalized MG; mRNA CAR‐T	Broad depletion of early and memory B cells; clinically meaningful improvement observed	Inability to eliminate long‐lived CD19⁻ plasma cells may permit relapse	Low‐grade CRS (grade 1–2); no ICANS reported	Granit et al. [53]
	BCMA	Case reports in refractory MG showing sustained clinical and serologic remission	Direct targeting of antibody‐secreting plasma cells; potentially more durable effect	BCMA is not universally expressed in all B lineage compartments; off‐tumor effects unclear	Only mild CRS; no ICANS observed	Tian et al. [52]
	CAAR‐T (AChR)	Preclinical stage; murine models targeting AChR‐specific B cells	Antigen‐specific deletion of autoreactive B cells; theoretically minimizes global immunosuppression	No clinical data; complex manufacturing and validation required	Preclinical only; safety not yet evaluated in humans	Konitsioti et al. [64]
NMOSD	CD19	Not yet studied in clinical trials; potential based on CD19⁺ memory B cell involvement	Targets precursor B cells responsible for AQP4‐IgG production	Fails to deplete CD19⁻ long‐lived CNS plasma cells	Not reported	Konitsioti et al. [64]
	BCMA	Phase 1 trial (CT103A) in AQP4⁺ NMOSD patients; relapse‐free survival achieved	Selective depletion of plasma cells producing AQP4‐IgG; rapid antibody titer decline	Requires further validation in larger cohorts; optimal dosing remains undefined	Mild CRS in most cases; no ICANS reported	Qin et al. [63]
	CAAR‐T (AQP4)	No preclinical or clinical data currently available	Antigen‐specific targeting of AQP4‐reactive B cells theoretically attractive	Lack of known AQP4 CAAR constructs; technical feasibility uncertain	—	—
MS	CD19	Early‐phase trials (KYV‐101); case reports demonstrate CSF CAR‐T penetration and symptom relief	Targets intrathecal B cells; reduces compartmentalized inflammation and antibody production	May not impact CNS‐resident CD19⁻ plasma cells; relapses may recur	Grade 1 CRS; no ICANS reported	Rankin, Shah [71]
	BCMA	Being evaluated in basket trials including MOG‐IgG diseases; no MS‐specific data yet	Depletes antibody‐secreting cells; rationale for progressive MS forms involving humoral immunity	Lack of MS‐specific trials; limited understanding of BCMA expression in CNS lesions	Not yet reported in MS	Konitsioti et al. [64]
	CAAR‐T (e.g., MOG)	Still in preclinical development; limited to in vitro or murine models	Enables precision killing of autoantigen‐specific B cells; preserves global immune function	High cost, design complexity, limited target availability	Preclinical; no human safety data	Konitsioti et al. [64]

### 5.4 Safety and Practical Limitations of CAR‐T in Neuroautoimmune Disease

#### 5.4.1 Incidence and Clinical Features of CRS and ICANS

CAR‐T therapy in hematologic malignancies consistently induces CRS in 57%–90% of patients, with severe (Grade ≥ 3) CRS in approximately 13%–32% of cases [82]. ICANS occurs in 20%–70% of treated individuals, with 11%–41% experiencing Grade ≥3 neurotoxicity [83]. Clinical manifestations range from mild delirium and aphasia to seizures or fatal cerebral edema.

Early phase trials of CD19 and BCMA CAR‐T in CNS autoimmune diseases (such as MG, NMOSD, and MS) reported only low‐grade CRS and rarely to no ICANS [84]. However, long‐term and large‐scale data are still lacking, underscoring the need for enhanced safety vigilance as these therapies approach broad clinical application.

#### 5.4.2 Mechanisms of CRS and ICANS: Risk Determinants

##### 5.4.2.1 CRS Pathophysiology

Triggered by CAR‐T cell activation and proliferation, leading to T‐cell release of IFN‐γ, which activates macrophages and endothelial cells, resulting in elevated IL‐6 and IL‐1, systemic inflammation, capillary leak syndrome, and organ dysfunction [85].

##### 5.4.2.2 ICANS Mechanism

Severe CRS typically occurs in cytokines such as IL‐6, GM‐CSF, and IFN‐γ, which disrupt the blood–brain barrier, allowing peripheral cytokines to activate microglia, causing neurotoxicity ranging from confusion to cerebral edema.

The risk factors included the following:

1. High CAR‐T cells dose‐dependently elevate CRS/ICANS.
2. Costimulatory domain design: CD28 constructs induce more rapid expansion and earlier onset of toxicity than 4‐1BB costimulation [83].
3. Lymphodepletion regimen: Intensive conditioning enhances T‐cell proliferation and cytokine release.
4. Disease burden, CNS status, high antigen load, and pre‐existing BBB compromise increase the risk of neurotoxicity [86].

In neuroautoimmune CAR‐T settings, these risks warrant cautious dose titration, selection of lower toxicity designs, and rigorous patient assessment [85].

#### 5.4.3 Dose Optimization and Trial Design Strategies

To reduce this risk, neuroautoimmune CAR‐T protocols should incorporate the following:

#### 5.4.4 Fractionated Dosing

Administering smaller aliquots over several days allows for early CRS/ICANS detection before the full‐dose delivery. Statistical dose‐escalation designs (such as TITE‐Steering) facilitate real‐time toxicity monitoring [85, 86].

##### 5.4.4.1 Low‐Activity CAR Constructs

Approaches such as low‐affinity targeting scFv (“affinity tuning”) have shown reduced CRS rates, while maintaining efficacy [83].

##### 5.4.4.2 Transient CAR Formats

mRNA‐based CARs express transiently, providing self‐limited exposure, an appealing model for autoimmune settings [84].

##### 5.4.4.3 Real‐Time Monitoring

Baseline and serial assessments, including cell‐associated encephalopathy (ICE) scores, cytokine panels (IL‐6, IL‐1, GM‐CSF), and CSF biomarkers (GFAP, NFL), enhance early detection and management.

#### 5.4.5 Safety Switches and Control Mechanisms

Suicide and control switches significantly enhance the safety of CAR‐T therapy:

##### 5.4.5.1 iCaspase‐9 (iCasp9)

AP1903 administration triggers rapid CAR‐T cell apoptosis. Phase 1 trials have revealed clearance within 1–2d [87].

##### 5.4.5.2 HSV‐TK/Ganciclovir

Virus‐derived, creates immunogenicity risk; therefore, slower action limits are used where rapid control is needed.

##### 5.4.5.3 Antigen‐Targeted Elimination Switches

Approaches such as the CD20–rituximab system (CubiCAR) enable antibody‐mediated control [88].

##### 5.4.5.4 Pharmacologic “On/Off” Systems

Dasatinib can transiently halt CAR signaling; adapter‐based logic switches using bispecific molecules allow external titration [89].

##### 5.4.5.5 Affinity Tuning

Reducing scFv affinity lowers the activation threshold, decreasing off‐target and neurotoxic activation (Table 2).

**Table 2:** Summary comparison.

Switch type	Advantages	Disadvantages
iCasp9 suicide switch	Fast, effective CAR clearance	Irrevocable CAR loss
Antibody‐mediated switch	Targeted control via mAb	Requires repeat dosing
On/Off pharmacologic switch	Reversible and dose‐titratable	Dependent on drug availability
Affinity‐tuning constructs	Low baseline cytokine risk	Requires precise CAR design

#### 5.4.6 CRS/ICANS Management Plan

Effective management of CAR‐T cell‐associated toxicities, such as CRS and ICANS, is essential, particularly for neuroautoimmune indications. CRS should be managed according to the ASTCT guidelines, with tocilizumab recommended for Grade ≥2 cases and short‐term corticosteroids added if the condition proves refractory [83, 87, 90]. For ICANS, the ICE scoring system is widely used; dexamethasone is initiated for Grade ≥2 events, with escalation to high‐dose corticosteroids and neurocritical care consultation in the presence of seizures or cerebral edema. Routine laboratory monitoring, including daily CBC, CRP, and IL‐6 level assessments, along with frequent neurological assessments and imaging (MRI and EEG), is essential for early detection. In addition, the use of anti‐IL‐1 agents, such as anakinra, is supported by emerging data in cases of severe CRS or neurotoxicity [83].

Moreover, the manufacturing, logistical, and economic challenges associated with CAR‐T cell implementation in autoimmune settings remain significant. Autologous CAR‐T cell therapies require 2–4 weeks of production time, which may be prolonged to 6 weeks because of logistical or pandemic‐related delays, a timeframe that is still tolerable in the context of chronic autoimmune disease [91]. The per‐dose cost of CAR‐T cells in hematological cancers typically ranges between USD 300,000 and 450,000 (SGD 400,000–600,000), raising questions regarding financial sustainability and insurance coverage for nonmalignant indications. Furthermore, infrastructure requirements, such as GMP‐grade manufacturing, cold‐chain logistics, and ICU‐level support for neurotoxicity management, significantly restrict the availability of CAR‐T therapies to a limited number of specialized centers.

Several strategies have been proposed to overcome these limitations. Allogeneic “off‐the‐shelf” CAR‐T products allow immediate availability and scalable manufacturing, although safety concerns, such as graft‐versus‐host disease (GvHD), necessitate strict controls [91]. In vivo CAR‐T engineering using mRNA or nanoparticle delivery systems provides a potential method to bypass ex vivo cell manipulation and reduce costs and turnaround time, although such approaches remain investigational [83]. Regional GMP manufacturing hubs embedded in academic medical centers can enable centralized production and economies of scale. Finally, value‐based payment models, where reimbursement is tied to clinical outcomes (as piloted with Kymriah and Yescarta), may provide a sustainable financing approach, although adaptation will be required in chronic autoimmune diseases that often necessitate long‐term or repeat dosing [92].

### 5.5 AE

#### 5.5.1 Rationale

AE is a group of immune‐mediated inflammatory disorders that primarily affect the brain, meninges, and spinal cord, with an annual incidence of approximately 1 per 100, 000 individuals. AE accounts for 10%–20% of encephalitis cases and typically manifests as subacute seizures, cognitive impairment, and psychiatric symptoms. Standard treatments, including steroids, intravenous immunoglobulin (IVIG), and plasmapheresis, often provide only partial relief; however, refractory cases remain a clinical challenge [93].

B cells play a central role in AE pathogenesis by producing autoantibodies that target neuronal surface or intracellular antigens. Emerging evidence has shown that CD19‐CAR‐T and BCMA‐CAR‐T may provide a focused approach by depleting autoreactive B cells in both peripheral and CNS compartments [65, 94].

#### 5.5.2 Current Clinical Evidence

Case reports and early trials involving anti‐CD19 and anti‐BCMA CAR‐T in CNS autoimmune disorders (such as NMOSD, MG, and stiff‐person syndrome) have reported manageable safety profiles and clinical improvement, although data on pure AE remain limited [64]. For instance, a patient with stiff‐person syndrome achieved sustained symptom improvement after anti‐CD19 CAR‐T therapy without reported ICANS or CRS. Large controlled trials specifically for AE are not yet available, underscoring the need for cautious optimism.

#### 5.5.3 Preclinical Data and Limitations

In EAE mouse models, a proxy for human AE, anti‐CD19 CAR‐T cells administered after cyclophosphamide reduced clinical severity and B‐cell infiltration in the CNS [78]. However, earlier study has indicated potential disease exacerbation in similar EAE settings, highlighting context‐dependent effects and the need for a precise CAR design [78]. These findings do not directly translate to human disease, and additional preclinical studies are required to optimize safety, dosing, and antigen specificity.

#### 5.5.4 Safety Profile

Although no serious neurotoxicity (ICANS) has been reported in a limited number of AE‐related CAR‐T cell cases, broader safety data remain insufficient. Concerns include the risk of broad B cell depletion, leading to immunodeficiency, off‐target effects, and long‐term consequences for the CNS immune environment [64].

### 5.6 Idiopathic Inflammatory Myopathy (IIM)

#### 5.6.1 Clinical Context and Rationale

IIMs are rare autoimmune conditions characterized by chronic muscle inflammation, weakness, and systemic complications. Despite treatment with glucocorticoids and immunosuppressants, patients often experience incomplete remission and serious side effects [95, 96]. CAR‐T therapy provides a targeted strategy by potentially eliminating autoreactive B cells with few off‐target effects.

#### 5.6.2 Preclinical Insights (Hypothesis Stage)

Preclinical CAR‐T constructs, including IL‐12 or IL‐18 secreting CAR‐T cells, show enhanced growth and persistence in autoimmune and cancer models, even without preconditioning  [97, 98]. However, none of these have been thoroughly evaluated in IIM mouse models or specifically addressed muscle inflammation in humans. These data support this concept but are not disease‐specific.

#### 5.6.3 Clinical Case Reports

Clinical evidence supports the therapeutic potential of CAR‐T cells in IIM and related autoimmune disorders. CD19‐targeted CAR‐T cells have been significantly successful in treating refractory anti‐SRP necrotizing myopathy, severe myositis, and systemic sclerosis, achieving sustained B‐cell depletion and significant clinical remission [99]. In a groundbreaking study, CRISPR‐Cas9‐engineered CD19‐directed CAR‐T cells derived from healthy donors were used to treat patients with refractory immune‐mediated necrotizing myopathy. The infused cells persisted for over 3 months, achieved complete B‐cell depletion within 2 weeks, and led to significant remission with no serious adverse events during the 6‐month follow‐up [100]. These findings highlight the potential of CAR‐T cells to induce durable remission with a favorable safety profile compared with conventional therapies [99].

An allogeneic CD19 CAR‐T (“TyU19”) is currently evaluated in IIM and systemic sclerosis, showing sustained peripheral B‐cell aplasia and a favorable safety profile at 6 months (NCT06462144). Additional universal CAR‐T constructs (such as RD06‐04) are in the early clinical stages of IIM and ANCA‐associated vasculitis, supporting the growing interest and feasibility of applying CAR‐T to inflammatory myopathies [1].

#### 5.6.4 Safety Considerations and Limitations

To date, reported adverse events are limited to low‐grade CRS; neither neurotoxicity (ICANS) nor opportunistic infections have been observed  [99, 100]. However, these data are limited to early phase studies without long‐term monitoring. Critical knowledge gaps remain regarding optimal CAR persistence, dosing strategies, immune reconstitution, relapse risk, and potential long‐term immune consequences.

### 5.7 Myelin Oligodendrocyte Glycoprotein Antibody‐Associated Disease (MOGAD)

#### 5.7.1 Disease Overview and Treatment Limitations

MOGAD is a rare autoimmune demyelinating disorder of the CNS that presents as optic neuritis, transverse myelitis, or acute disseminated encephalomyelitis [101, 102]. Diagnosis primarily depends on MOG‐IgG detection using cell‐based assays and MRI findings [101]. Acute episodes are treated with high‐dose corticosteroids and plasma exchange, followed by immunosuppressants such as azathioprine, mycophenolate mofetil, or rituximab to prevent relapse. IVIG reduces the relapse frequency [103]. However, some patients remain refractory to current therapies, highlighting the need for novel therapeutic approaches.

#### 5.7.2 CAR‐T Therapy: Emerging Evidence and Case Insights

CAR‐T therapy, a breakthrough in oncology, is currently being explored for the treatment of autoimmune disease. In EAE, a common demyelination model, CD19‐targeted CAR‐T cells effectively deplete B cells in both the peripheral and CNS compartments, revealing their potential efficacy in MOGAD [78]. A recent case report described an 18‐year‐old patient with treatment‐resistant MOGAD who achieved remission following CD19‐CAR‐T therapy with no major adverse effects [104]. Although this is an isolated case, it illustrates a promising therapeutic direction for refractory MOGAD.

#### 5.7.3 Next‐Generation CAR‐T Technologies

Innovations in CAR‐T cell design may further enhance their suitability for treating CNS autoimmune diseases. Synthetic Notch CAR‐T cells, originally designed for glioblastoma, provide improved targeting with few off‐target effects, making them candidates for MOGAD treatment under similar conditions [105]. In addition, liver‐directed gene therapy, which induces MOG‐specific Treg cells, has shown symptom reversal in animal models, providing a complementary immunomodulatory strategy [81].

#### 5.7.4 Challenges and Future Directions

Despite the encouraging preliminary data, CAR‐T therapy for MOGAD remains largely under investigation. The rarity of the disease limits large‐scale clinical trials, and patient heterogeneity necessitates individualized approaches. Key areas for future research include optimizing CAR design, minimizing toxicities such as CRS, and ensuring long‐term efficacy and safety [75, 106].

### 5.8 Guillain–Barré Syndrome (GBS)

#### 5.8.1 Disease Overview and Treatment Limitations

GBS is an acute immune‐mediated peripheral neuropathy characterized by rapidly progressive weakness and areflexia. Although plasma exchange and IVIG remain the cornerstones of treatment, patients often experience incomplete recovery, highlighting the need for alternative therapeutic strategies [107].

#### 5.8.2 CAR‐T Therapy: Immunomodulatory Potential

Originally developed for hematologic malignancies, CAR‐T therapy can selectively target pathogenic immune cells. In the context of GBS, the theoretical utility of CAR‐T lies in the depletion of autoreactive B cells and reduction of pathogenic autoantibodies involved in disease progression [108, 109].

#### 5.8.3 Safety Concerns and Mechanistic Complexity

Emerging case reports have documented rare instances of CAR‐T cell‐induced Guillain–Barré‐like syndromes, underscoring the complex interplay between CAR‐T cells and peripheral immune regulation [110]. Moreover, known adverse events, such as CRS and ICANS, warrant careful monitoring [108, 109]. Biomarkers, such as ferritin levels and platelet counts, may aid in predicting the severity of neurotoxicity and enhancing clinical safety [111].

#### 5.8.4 Future Directions and Research Needs

Further research is essential to define the role of CAR‐T cells in GBS, both as a therapeutic innovation and as a potential trigger for immune complications. Optimization of antigen targeting, mitigation of adverse effects, and a deep understanding of GBS pathogenesis are key to translating CAR‐T cells into viable treatment options [112] (Table 3).

**Table 3:** All diseases: comparison and summary.

Disease	CAR‐T target	Efficacy	Safety	Clinical trial phase	Clinical Trial ID	Commercial availability	Trial completion status	Reference
Myasthenia gravis (MG)	CD19, BCMA, MuSK	Reduced AChR autoantibodies, sustained remission	Favorable profile, no dose‐limiting toxicities	Phase 1/2	NCT04146051	No	Phase1	Haghikia et al. [49, 50, 52]
Neuromyelitis optica spectrum disorder (NMOSD)	BCMA, CD19, AQP4‐IgG	Significant clinical improvement; reduced AQP4‐IgG levels	Minimal CRS and neurotoxicity	Phase 1	NCT06561009, NCT06633042	No	Phase1	[60, 63, 65]
Multiple sclerosis (MS)	CD19, Myelin‐specific CARs	Reduction in antibody production, modulation of immune response	No ICANS observed; CNS safety demonstrated	Phase 1	NCT06220201	No	Phase1	[70, 71, 78]
Autoimmune encephalitis (AE)	CD19, BCMA	Effective in refractory cases, symptom alleviation	Favorable with no significant ICANS	Phase 1/2	NCT04146051	No	Phase1	[65, 78, 94]
Idiopathic inflammatory myopathy (IIM)	CD19, Enhanced CAR‐T designs	Significant remission in refractory cases	Favorable with reduced CRS risk	Phase 1	NCT06462144	No	Phase1	[95, 99, 100]
Myelin oligodendrocyte glycoprotein antibody‐associated disease (MOGAD)	CD19	Clinical improvement in refractory cases	Safety demonstrated in case studies	Case study	Case Report	No	Completed (Case Study)	Cabrera‐Maqueda et al. [101, 103, 104]
Guillain–Barré Syndrome (GBS)	Autoreactive B cells	Potential reduction in autoantibody production	Safety data limited; potential adverse events	Exploratory	N/A	No	N/A	Brudno, Kochenderfer [108, 110, 112]

## 6 Innovations in CAR‐T Therapy

### 6.1 Targeting Autoreactive T Cells With CAR‐T Therapy

Although most current clinical applications of CAR‐T therapy in autoimmune diseases have concentrated on eliminating autoreactive B cells, a growing body of evidence indicates that pathogenic T cells play a central role in driving neuroimmunological disorders, such as MS and AE. Thus, directly targeting autoreactive T cells represents a complementary and potentially transformative therapeutic approach [4].

CD7 is a transmembrane glycoprotein expressed on the majority of mature T cells and subsets of NK cells, making it an attractive antigen for the depletion of pathogenic T‐cell clones. CD7‐directed CAR‐T cells have been extensively investigated in T‐cell malignancies and have shown encouraging efficacy with manageable safety [113]. This clinical experience highlights the feasibility of extending CD7‐CAR‐T therapy to autoimmune diseases, in which pathogenic T cells sustain chronic inflammation and tissue injury.

#### 6.1.1 Key Challenges of T‐Cell‐Directed CAR‐T Therapy

A major technical challenge is fratricide, whereby CAR‐T cells kill each other due to endogenous CD7 expression, impairing their expansion and persistence [114]. In addition, broad depletion of CD7^+^ T cells may result in T‐cell aplasia, leading to an increased infection risk and the need for immune reconstitution strategies [115]. Moreover, product contamination by malignant or autoreactive T cells during manufacturing represents a safety concern [113].

#### 6.1.2 Innovative Strategies to Overcome Fratricide and Improve Safety

Several approaches have been developed to mitigate these challenges:

1. Gene or base editing to delete CD7 (or related loci) before CAR introduction prevents fratricide and enables robust CAR‐T expansion [116].
2. Protein expression blockers or natural selection strategies suppress CD7 surface expression or enrich CD7‐low subsets during manufacturing, avoiding fratricide without permanent genome modification [117].
3. Pharmacologic blockade of CAR signaling (such as dasatinib “on/off switches”) during production temporarily prevents fratricide and preserves functional CAR‐T cell output [118].
4. Allogeneic “off‐the‐shelf” CD7‐CAR‐T products incorporating multi‐locus editing (such as TRAC/CD7/CD52 knockout) allow scalable manufacturing while reducing GvHD risk [116].
5. Selective targeting of T‐cell receptor (TCR) β‐chain constant regions (TRBC1/2) provides a more refined strategy to eliminate clonally expanded pathogenic T cells while sparing normal repertoires [119].

#### 6.1.3 Clinical Insights and Future Perspectives

Early phase clinical trials of T‐cell malignancies have shown high response rates with CD7‐CAR‐T cells, although infectious complications and relapse remain a concern [115, 120]. In the field of autoimmunity, direct clinical evidence remain scarce, with most success to date derived from B‐cell‐directed CAR‐T therapies (such as CD19 and BCMA). However, preclinical studies have strongly indicated that CD7‐CAR‐T cells can reduce autoreactive T‐cell populations, ameliorate disease severity, and promote durable immune tolerance. Moving forward, careful trial designs with stringent immune monitoring, infection prophylaxis, and long‐term immune reconstitution strategies will be essential. Moreover, combining T‐cell‐targeted (such as CD7) and B‐cell‐targeted CAR‐T approaches may provide synergistic benefits by simultaneously modulating both cellular and humoral arms of autoimmunity.

### 6.2 Allogeneic CAR‐T Therapy: Strategies for TCR and HLA‐I/II Knockout

The development of allogeneic CAR‐T therapy (“off‐the‐shelf” CAR‐T) aims to overcome the limitations of autologous CAR‐T, including lengthy manufacturing time, product variability, and challenges in heavily pretreated patients or patients with lymphopenia. However, the use of donor‐derived T cells introduces the risk of GvHD mediated by endogenous TCR recognition, and host‐versus‐graft rejection driven by HLA mismatches. To address these challenges, sophisticated genome‐editing strategies have been applied to disrupt TCR and HLA expression [121, 122].

#### 6.2.1 TCR Knockout

Eliminating the expression of the TCR α‐chain (TRAC) gene through CRISPR/Cas9 or TALEN prevents T cells from recognizing host alloantigens, thereby significantly reducing the risk of GvHD. TRAC knockout has become a standard modification in most allogeneic CAR‐T pipelines and has been validated in multiple clinical trials [123].

#### 6.2.2 HLA Class I Knockout

Disruption of the β2‐microglobulin (B2M) gene abolishes surface HLA class I expression, rendering donor CAR‐T cells resistant to cytotoxic CD8^+^ T cell‐mediated rejection. However, the absence of HLA class I may render cells vulnerable to NK cell‐mediated killing. To address this, several groups have introduced non‐polymorphic HLA molecules, such as HLA‐E or HLA‐G, to inhibit NK cell cytotoxicity [123].

#### 6.2.3 HLA Class II Knockout

Targeting the CIITA gene, a transcriptional activator of HLA class II, prevents the upregulation of class II molecules during T‐cell activation and thereby reducing recognition by host CD4^+^ T cells [123].

#### 6.2.4 Combination Editing Strategies

Recent clinical studies have reported the feasibility and safety of “multiplex‐edited” allogeneic CAR‐T cells incorporating TRAC, B2M, and CIITA knockout simultaneously. These next‐generation products are designed to minimize GvHD, reduce host rejection, and enhance persistence in vivo [124]. Early phase clinical trials of hematologic malignancies have shown that such genome‐edited allogeneic CAR‐T therapies can induce durable remissions with manageable safety profiles.

Overall, allogeneic CAR‐T cells modified through TCR and HLA‐I/II knockout represent a promising step toward developing universal CAR‐T products. For autoimmune and neuroimmunological disorders, these advances may enable scalable, standardized therapies, thereby facilitating broader patient access and faster clinical application [125].

### 6.3 In Vivo CAR Engineering: Emerging Applications in Autoimmune Disease

In vivo CAR engineering has recently emerged as an innovative strategy to address several limitations of conventional ex vivo CAR‐T therapy, such as prolonged manufacturing time, high cost, variable product quality, and the requirement for lymphodepleting chemotherapy. By directly reprogramming T cells inside the patient, this approach bypasses the complexities of ex vivo cell manipulation and may enable more rapid, scalable, and accessible immunotherapies [126, 127, 128].

#### 6.3.1 Recent Breakthroughs

Two landmark studies published in 2025 highlighted the potential of in vivo CAR engineering for cancer and autoimmune disease.

- Hunter et al. [126] developed targeted lipid nanoparticles (tLNPs) encapsulating mRNA encoding an anti‐CD19 CAR. These nanoparticles specifically delivered CAR constructs to circulating CD8^+^ T cells in vivo. In humanized mouse models, engineered T cells expand and mediate B‐cell depletion. In nonhuman primates, systemic administration achieved robust B‐cell depletion followed by reconstitution with predominantly naïve B cells, revealing an immune “reset.” When applied to T cells derived from patients with autoimmune diseases, tLNPs successfully redirected T cells to deplete B cells, providing translational evidence for autoimmune therapy (PubMed link).
- Li et al. [127] provided a comprehensive review of in vivo CAR engineering platforms, including mRNA‐based nanoparticles, viral vectors, and implantable bioinstructive scaffolds. The authors compared in vivo and ex vivo approaches in terms of efficacy, persistence, safety, and feasibility, while emphasizing unique challenges, such as targeting specificity, controlling the duration of CAR expression, immunogenicity of delivery vehicles, and risks of off‐target editing. They highlighted the promise of in vivo CAR engineering for autoimmune diseases, in which short‐lived but potent immune “reprogramming” may suffice to deplete autoreactive lymphocytes without inducing long‐term immunosuppression (PubMed link).

#### 6.3.2 Advantages and Challenges in Autoimmune Disease Context

- Advantages: (i) circumvents ex vivo manufacturing, reducing time and cost; (ii) allows rapid reprogramming of endogenous T cells; (iii) transient CAR expression through mRNA may limit long‐term adverse effects; (iv) potential to induce immune reset by selectively depleting autoreactive B or T cells [128].
- Challenges: (i) ensuring selective targeting of appropriate T‐cell subsets; (ii) balancing expression strength and duration (transient vs. long‐term); (iii) potential immunogenicity of nanoparticle or viral delivery platforms; (iv) ensuring safety in terms of biodistribution and avoiding off‐target effects; (v) translating promising animal model findings to human autoimmune conditions [126].

#### 6.3.3 Future Perspectives

In vivo CAR engineering holds great promise for treating neuroimmunological and systemic autoimmune diseases. By enabling rapid, patient‐specific CAR‐T generation, this approach may broaden access and reduce barriers to therapy. Moreover, the ability to fine‐tune CAR persistence and combine in vivo reprogramming with next‐generation safety switches could provide a safer and more adaptable therapeutic platform. Future clinical studies will need to address key issues such as dosing, delivery route, immune monitoring, and durability of response, while carefully evaluating the risks of immunogenicity and long‐term safety [126, 129].

### 6.4 Miscellaneous

The development of CAR‐T cell therapies for neuroimmunological disorders is progressing rapidly, with a specific emphasis on enhancing their effectiveness and safety. One promising strategy is to target specific antigens, as seen in MG, where CAR‐T therapy targeting B cells associated with anti‐MuSK antibodies effectively reduces autoantibody production and inflammation, leading to significant clinical improvement [43]. This method underscores the ability of CAR‐T cells to selectively target disease‐specific immune cells without inducing widespread immunosuppression.

Another novel approach entails the use of CAR‐Tregs engineered to reestablish immune tolerance and mitigate immune‐driven damage in conditions such as MS and AE. By introducing CAR‐Tregs, researchers aim to suppress the pathological immune responses that drive tissue damage and promote long‐term remission in these conditions. Clinical trials have shown promising results regarding the ability of CAR‐Tregs to modulate immune responses without exacerbating systemic immunosuppression, thereby providing a safer alternative to conventional immunosuppressive treatments.

In addition, multi‐targeted CAR‐T cells are being developed to overcome antigen escape, a phenomenon in which tumor or immune cells evolve to evade the targeted therapies. By targeting multiple antigens simultaneously, CAR‐T cells ensure continued therapeutic efficacy in complex diseases with heterogeneous antigens, such as relapsed or refractory cancers and neuroimmunological diseases [130]. For example, dual‐target CAR‐T cells that target both CD19 and CD22 have shown efficacy in reducing antigen escape and improving outcomes in patients with relapsed/refractory acute lymphoblastic leukemia [131].

Recent advancements include the use of CRISPR/Cas9 gene editing and RNA engineering to enhance CAR‐T therapy efficacy. CRISPR technology allows for precise genomic modifications and optimization of CAR‐T cells to address challenges such as T cell exhaustion, toxicity, and manufacturing limitations [132]. For instance, CRISPR/Cas9 can be used to create CAR‐T cells with enhanced persistence and reduced susceptibility to exhaustion, improving their long‐term efficacy in neuroimmunological diseases. RNA‐based methodologies are emerging as a means to regulate CAR‐T cell activity in vivo, enabling precise adjustment of their therapeutic effects and reducing adverse effects, such as CRS and neurotoxicity [133]. For example, RNA‐engineered CAR‐T cells have shown promise in reducing CRS in clinical trials, thereby providing a safer therapeutic alternative.

Moreover, synthetic biology is being explored to develop genetic switches that provide spatiotemporal control over CAR‐T activity, enhance safety by preventing uncontrolled immune responses, and minimize off‐target effects [85].

Another crucial area of innovation focuses on reducing CAR‐T cell manufacturing time, which traditionally takes 2–4 weeks from leukapheresis to infusion for autologous products. To accelerate this timeline, semi‐automated, closed‐system manufacturing platforms, such as the CliniMACS Prodigy system, are being implemented in early phase clinical settings to allow on‐site, decentralized CAR‐T production within days instead of weeks [134]. In addition, mRNA‐based CAR‐T cells, which do not require genomic integration, have a faster production cycle (within 1–2 days) and are currently being evaluated for transient and safe CAR expression in autoimmune disease contexts [135].

Allogeneic CAR‐T cells (allo‐CAR‐T) have been actively investigated to overcome the logistical and cost barriers of individualized autologous therapies. They are derived from healthy donors and can be manufactured in bulk and cryopreserved as “off‐the‐shelf” therapies, enabling immediate availability and reduced cost per dose [136]. Allo‐CAR‐T has shown promising efficacy in hematological malignancies, with manageable risks of GvHD when genome‐editing tools (such as TALEN or CRISPR) are used to eliminate endogenous TCR expression [137]. The feasibility of using allo‐CAR‐T cells in autoimmune CNS diseases is currently being explored in preclinical models and early phase trials, particularly in settings requiring rapid intervention or in patients with lymphopenia or manufacturing failure.

Despite these advances, several challenges remain. For example, immune tolerance in neuroimmunological diseases poses a unique challenge because as the immune system must be carefully modulated without compromising the ability of the body to respond to infections. In MS, CRS may develop after CAR‐T therapy, resulting in CNS inflammation and potentially worsening disease. Mitigating these challenges may involve the use of combination treatments, such as integrating CAR‐T therapy with immunomodulatory drugs or checkpoint inhibitors, to enhance both safety and effectiveness. Customized CAR‐T therapy, designed to address patient‐specific antigens and immune responses, has the potential to overcome these challenges by providing targeted treatment alternatives. This approach could be particularly beneficial in the context of neuroimmunological diseases, where the precise timing of immune modulation is critical to avoid damaging healthy CNS tissues [138, 139].

Although these innovations in CAR‐T therapy are still in their early stages, they hold significant promise for treating neuroimmunological disorders. By overcoming current limitations, such as immune tolerance and CRS, and leveraging advanced technologies, such as CRISPR and RNA engineering, CAR‐T therapies have the potential to revolutionize the treatment of diseases, such as MS, AE, and other CNS autoimmune diseases. As research continues, combination therapies and precision medicine approaches will be key to optimizing these therapies and expanding their applicability [138, 139]. The future of CAR‐T therapy lies in harnessing these innovations to provide personalized, precise, and effective treatments for patients with complex neuroimmunological diseases [140].

## 7 Conclusion

CAR‐T therapy has emerged as a powerful immunotherapeutic tool for treating hematological malignancies and autoimmune diseases. In oncology, targeted tumor antigen recognition has revolutionized the treatment of conditions such as B‐cell acute lymphoblastic leukemia and non‐Hodgkin lymphoma. In neuroimmunological diseases, such as MG, NMOSD, and MS, CD19‐, BCMA‐, and MuSK‐targeted CAR‐T cells have shown promise in depleting pathogenic B cells and restoring immune balance in refractory cases.

However, clinical evidence remains limited for other autoimmune conditions, such as GBS and MOGAD, for which CAR‐T therapy is still in its early stages. Therefore, large multicenter trials are required to establish the long‐term safety and efficacy.

Future research should focus on optimizing CAR‐T cell design, including dual‐targeting strategies and integration with immune modulators, to enhance precision and reduce adverse events such as CRS and neurotoxicity. Personalized CAR‐T therapies tailored to individual immunopathologies and supported by advances in synthetic biology may further improve safety and therapeutic outcomes.

CAR‐T therapy has transformative potential for autoimmune diseases and is likely to become a cornerstone of personalized immunotherapy in the near future.

## Author Contributions

Y.P. received the funding support and developed the research hypotheses. Y.P., Y.S.Y., M.Q.D., H.Y., Q.M.Z., H.Z., X.L.Z., S.K., J.L., L.X.L., X.H.K., and D.Y. wrote the manuscript. The final manuscript is the end product of the joint writing efforts of all authors.

## Ethics Statement

The authors have nothing to report.

## Consent

The authors have nothing to report.

## Conflicts of Interest

The authors declare no conflicts of interest.

## Data Availability

The authors have nothing to report.
